# Sleep does not occur in isolation: contextualizing adolescent sleep disparities

**DOI:** 10.1007/s44470-026-00201-9

**Published:** 2026-10-12

**Authors:** BreAnne Young, Azizi Seixas, Girardin Jean-Louis

**Affiliations:** Department of Psychiatry and Behavioral Sciences, University of Miami Miller School of Medicine, 1120 NW 14th Street, Miami, FL 33132 USA

Adolescence is a period of significant developmental change in which physical, social, and environmental demands converge with unusual intensity. Youth are in the midst of navigating earlier school schedules, extracurricular activities, family responsibilities, changes in their social networks, and greater digital engagement, all of which increasingly compete for finite sleep opportunity. Adolescence is also marked by a shift in circadian biology as the body enters puberty, leading to a shift in the release of melatonin that leaves most teens struggling to fall asleep before 11 pm [1]. Consequently, many youths begin the school day with insufficient sleep, potentially compromising alertness, readiness to learn, and school performance.

The study by Teferi et al. that was recently published in the *Journal of Clinical Sleep Medicine *provides an important exploration of whether sleep duration and chronotype explain racial/ethnic differences in academic outcomes among ninth-grade students in semirural Georgia [2]. While neither sleep dimension significantly mediated differences in grade point average (GPA) or absences, several findings emerged that reinforce sleep’s relevancy in adolescent scholastic performance. Specifically, longer sleep duration was associated with higher GPA, later chronotype was associated with greater absenteeism, and racial/ethnic differences were observed in GPA, absences, and chronotype. Together, these results suggest that sleep and circadian timing are associated with academic outcomes, but do not account for the broader landscape of social, economic, biologic, and institutional conditions that influence adolescent development and educational opportunity.

The distinction between mediation and association is central to this interpretation. Considerable evidence links adolescent sleep with attention, learning, memory, emotional regulation, cognitive functioning, and readiness to learn. At the same time, meta-analytic evidence suggests that academic performance can vary across sleep dimensions, with sleep quality showing more consistent association with academic outcomes than sleep duration alone [3]. More recent evidence similarly suggests modest overall associations between sleep and academic functioning, with variations presenting based on the sleep parameter examined and the socioecological circumstances in which young people live [4]. Thus, a null indirect effect for sleep duration may indicate that duration captures only one component of a multidimensional sleep health profile.

The specification of race and ethnicity within mediation models also warrants careful interpretation. Because race is a social construct rather than a manipulable biologic exposure, it reflects the socially patterned exposures, resources, and barriers that can arise through structural and interpersonal processes [4, 5]. Accordingly, racial/ethnic differences in academic outcomes may reflect unequal distributions of educational resources, economic conditions, discrimination, neighborhood environments, school climate, and other contextual influences. Within this framework, it is conceivable that sleep serves as one pathway through which these conditions affect adolescent functioning without statistically mediating the association between racial/ethnic classification and academic outcomes. Sleep could also serve as a moderator in this relationship, in which restorative sleep could strengthen an adolescent’s capacity to meet academic demands, and insufficient sleep could instead amplify these stressors.

Figure 1 organizes this interpretation into three interacting levels of influence: individual biology, the proximal lived environment, and the institutional environment. These contexts shape multiple dimensions of sleep health, which may affect academic outcomes through alertness, memory, executive function, mood, and readiness to learn. This wider framing may help explain why sleep can be highly consequential for adolescent health and functioning while showing only modest or inconsistent mediation of educational disparities [4, 5].

**Fig. 1:**
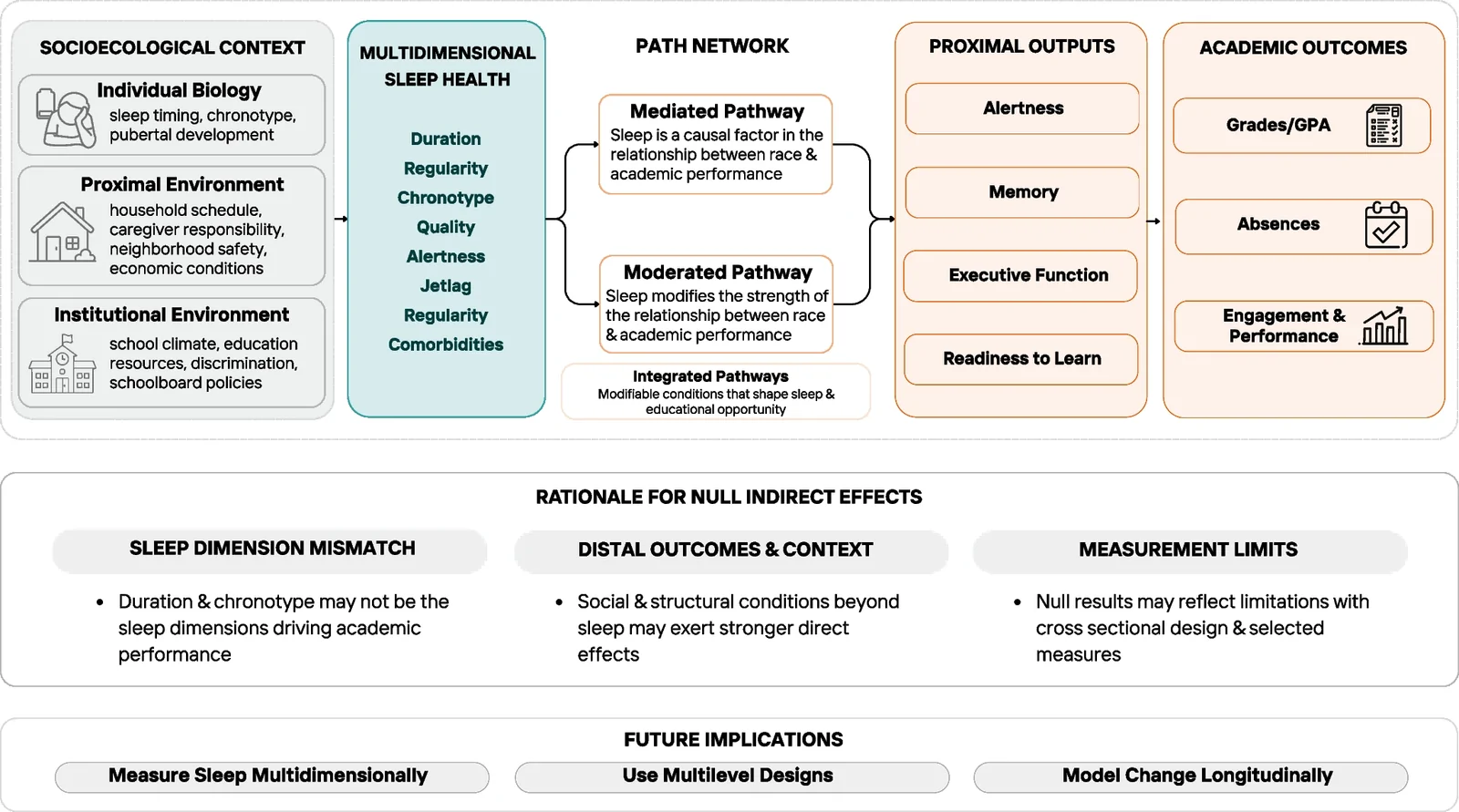
Contextual framework linking sleep health with academic outcomes. Individual biology, the proximal lived environment, and the institutional environment shape multidimensional sleep health. Sleep may mediate group differences or moderate vulnerability and resilience by amplifying or buffering adverse conditions. Alertness, memory, executive function, mood, and readiness to learn connect sleep with distal academic outcomes. This framework identifies three priorities for future research: multidimensional sleep measurement, multilevel contextual assessment, and longitudinal design.

The chronotype findings also merit similar consideration. The authors report that later chronotype was associated with greater absenteeism even though chronotype did not mediate racial/ethnic differences in absences. Adolescents naturally shift toward later circadian timing during puberty, but school schedules generally continue to require early wake times [6]. For evening-oriented adolescents, this mismatch can create circadian misalignment and lead to social jetlag, or misalignment between their biologically preferred sleep timing and socially required schedules. Recent studies have also linked social jetlag and later chronotype with poorer cognitive and academic performance; similarly, others report later school start times are associated with longer sleep and lower social jetlag [7, 8]. The observed association with absenteeism may therefore be a reflection of adolescents’ difficulty functioning within schedules that are poorly aligned with their circadian biology.

Teferi et al.’s study period further complicates this interpretation. It is important to note that the data in the present study were collected during the 2019–2020 academic year, coinciding with the onset of the COVID-19 pandemic and unprecedented disruptions to adolescents’ educational and social environments. School closures and remote learning altered daily schedules and sleep opportunity for many adolescents, often delaying wake times and changing weekday–weekend differences in sleep timing [5]. These disruptions may change how we interpret the sleep duration and chronotype outcomes observed during this period. Moreover, the absence of other sleep-related and contextual measures, in addition to the study’s cross-sectional design and reliance on self-reported sleep data limits the inferences we can make about sleep and educational outcomes.

These limitations point towards a more comprehensive framing of sleep health in which sleep serves as an interface through which social and educational environments influence adolescent functioning. Future studies should assess multiple dimensions of sleep, ideally using longitudinal designs and complementary objective and self-report measures. Testing how sleep duration, timing, regularity, and quality jointly interact with school, household, and neighborhood conditions may provide a clearer understanding of the role of racial/ethnic identity in adolescent sleep and academic performance. This distinction redirects the field from searching for one mediating variable towards testing a network of modifiable pathways.

Examined from this perspective, Teferi et al.’s null mediation highlights where our next inquiry in this public health area should begin. Sleep matters for academic functioning, but it does not occur in isolation. Moving forward, investigators must identify how sleep interacts with the environments in which adolescents live and learn, and examine multilevel approaches to improve restorative sleep and equitable opportunity.

## Data Availability

Not applicable.
